# What does attitude towards risk tell us about the adoption of good health behaviours? Results from a telephone survey

**DOI:** 10.1186/s12889-026-27960-7

**Published:** 2026-06-11

**Authors:** Christine Sevilla-Dedieu, Morgane Le Guern, Nathalie Billaudeau, Alain Paraponaris

**Affiliations:** 1 MGEN Foundation for Public Health, 3 square Max Hymans, 75748, Paris Cedex, France; 2 Aix Marseille Univ, CNRS, AMSE, Marseille, France

**Keywords:** Health behaviours, Sports, Tobacco, Weight, Attitude towards risk

## Abstract

**Background:**

The literature shows that the adoption of good health behaviours is positively linked to individual factors, such as educational or socioeconomic levels. In this study, we aim to explore the link between an individual’s attitude towards risk and the adoption of positive health habits (e.g., being sporty, not smoking or having a normal Body Mass Index – BMI –).

**Methods:**

We analysed survey data collected by phone from 2,666 individuals selected at random from a population of French health insurees. In addition to the individual’s socioeconomic characteristics (age, gender, marital status, number of children, level of education, financial difficulties) and health status, we specifically analysed the role of his/her score for risk taking, measured using the 30-item self-report DOSPERT scale, in the adoption of each good health behaviour, using logistic models.

**Results:**

Among the 2,666 respondents, 58.0% reported participating in sports regularly, 71.6% did not smoke, and 61.8% reported having a normal BMI. The logistic models show a strong significant association (*p* < 0.01) between the respondent’s DOSPERT risk score and his/her health habits; however, the sign depends on the behaviour considered.

**Conclusion:**

Our results reveal the complexity of the relationship, which is not unequivocal, between an individual’s attitude towards risk and the adoption of these good health behaviours. Health-conscious individuals do not exhibit a uniform pattern of behaviour: their choices regarding diet, physical activity and smoking, reflect distinct and sometimes inconsistent trade-offs.

**Supplementary Information:**

The online version contains supplementary material available at 10.1186/s12889-026-27960-7.

## Introduction

As defined by the World Health Organization (WHO), prevention consists of avoiding the appearance, development or worsening of diseases or incapacities. Acting on health behaviours and promoting vaccination can be considered the first steps of prevention, often called primary prevention. The purpose of primary prevention is to promote change in healthy people to prevent the onset of disease [1].

In this paper, we are interested in three so-called good health behaviours: practising sports regularly, not smoking and having a Body Mass Index (BMI) that is considered normal. A wealth of scientific literature shows that adopting such behaviours may contribute to reducing mortality in general [2] and may also be protective against several common chronic conditions, such as cancers [3–6], cardiovascular diseases [7–9] and diabetes [10–12]. The burden of these chronic diseases is far from negligible. It concerned 3.3 million people and caused an expense of 20.1 billion € (12.0% of the total healthcare expenditures reimbursed by the National Health Insurance – NHI –) in 2019 in France for cancers, respectively 13.6 million people and 23.5 billion € (14.1% of the total expense) for cardiovascular diseases and chronic treatments, and 4.0 million people and 8.6 billion € (5.2% of the total expense) for diabetes [13]. Finally, the public health issue is all the more obvious that those three health conditions represented more than half of all deaths which occurred in France in 2021 (25.7% for cancers, 20.9% for cardiovascular diseases and 3.6% for diabetes) [14].

Many factors that support the adoption of these behaviours, such as a higher educational or socioeconomic level, have been identified in the literature and are now common knowledge. According to recent publications, sedentariness is generally less common in women and in people who are younger, have more education or have a higher income [15]. In addition, recent research on tobacco highlights the negative association between smoking and either increasing age or a higher educational or socioeconomic level [16–18]. Finally, regarding overweight or obesity, recent studies have focused on socioeconomic, biological, behavioural and psychological factors, such as stress in childhood or in relation to life events [19–21]. The environment may also play a key role, particularly in terms of access to food, sports and health amenities [22, 23] or residence in a disadvantaged neighbourhood in a rich country [24–26]. Finally, the relationship between income and obesity has been found to depend on a country’s wealth and is significantly positive in poor countries [27].

The aim of this paper is to contribute to this literature by exploring a type of factors that has not been extensively studied, personal traits, and, more precisely, the possible association between an individual’s attitude towards risk and the adoption of risky/healthy behaviours. From a theoretical perspective, risk aversion is commonly defined as the preference for certain outcomes over uncertain ones with equal or higher expected value [28]. Within the expected utility framework, this trait is generally understood to motivate both insurance (reducing the financial consequences of adverse events) and prevention (reducing their probability), although early theoretical contributions have drawn a distinction between self-insurance and self-protection, sometimes suggesting that risk aversion plays a more limited role in the latter [29–31]. More recent economic approaches, however, emphasize that individuals allocate risk-reducing effort across multiple domains subject to heterogeneous costs, constraints, and adjustment frictions, implying that the relationship between risk attitudes and health behaviours is inherently ambiguous and potentially domain-specific. In particular, behaviours differ in their accessibility and in the magnitude of the behavioural change they require, which may lead risk-averse individuals to adopt some preventive actions while neglecting others. Empirically, however, the evidence remains limited and fragmented. To the best of our knowledge, only a small number of studies have directly examined the association between risk attitudes and health-related behaviours, and their findings point to a nuanced relationship rather than a uniform effect [32–34]. These contributions suggest that, despite mixed theoretical predictions, risk attitudes are significantly associated with both risky and protective behaviours, although the direction and magnitude of this relationship vary across behavioural domains. This relative scarcity of empirical evidence, combined with the theoretical ambiguity surrounding the role of risk aversion in prevention, underscores the need for further investigation.

To this end, the present study aimed to empirically describe the relationships between people’s attitudes towards risk and their adherence to the three health behaviours: diet, smoking and physical activity (which constitutes a behavioural domain through which risk preferences are expressed, notably via its links with physical risk-taking and individual endowments [35]), using survey data collected by phone from randomly selected individuals covered by a major French complementary health insurer. In our study, individuals’ attitude towards risk was measured by their DOSPERT score, which was calculated on the basis of their answers to the 30-item self-report Domain-Specific Risk-Taking (DOSPERT) scale using five dimensions: ethical, social, health/security, financial and recreational activities [36, 37]. In view of the literature, this scale is interesting for two reasons: (1) risk preferences elicited with a direct approach instead of lotteries may better predict risky behaviours [38], and (2) although they were observed within a different population (doctors), attitudes towards risk proved to be specific to each field rather than globally consistent, which justifies the use of multidimensional approaches to better account for the heterogeneity of health behaviours [39].

## Methods

### Sample

The survey sample was composed of insured adults, covered by a complementary health insurance (CHI) contract with the second-largest complementary insurer in France: MGEN, a French non-profit mutual insurance company originally established for education, research, and culture staff but now open to a broader population. In France, the CHI corresponds to an additional policy taken out by 95% of the population covered by the *Sécurité Sociale*, the NHI that provides universal health insurance [40].

Of the 4,580 policyholders selected at random, 2,757 agreed to take part in a telephone survey conducted between March and June 2019 for a participation rate of 61%. Our sample consists of the 2,666 insured persons who responded to the questions under study here. According to Table 1, 53% of these individuals were younger than 46 years old. They had 1.1 children on average. The majority of the participants had an education level of at least a baccalaureate level and a median level of CHI coverage (81.4% and 57.4%, respectively). A quarter of them (25.4%) had some financial difficulties.

**Table 1:** Characteristics of the respondents as a percentage or average (*N* = 2,666)

	Overall sample (*N* = 2,666)	Men (*N* = 1,217)	Women (*N* = 1,499)	*P* value
Sociodemographics				
Female	54.4%	-	-	-
Age				
Less than 36 years	25.4%	24.6%	26.1%	0.3951
36–45 years	27.6%	29.9%	25.7%	0.0147
46–65 years	25.5%	23.7%	27.1%	0.0413
Over 65 years	21.5%	21.8%	21.1%	0.6836
Living as a couple	49.6%	49.4%	49.8%	0.8195
Number of children	1.1	1.0	1.4	0.0000
No university degree^*^	18.6%	20.7%	16.8%	0.0105
Bachelor’s degree	15.0%	16.2%	14.1%	0.1290
Undergraduate diploma	37.4%	32.3%	41.7%	0.0000
Postgraduate diploma	29.0%	30.8%	27.4%	0.0532
Health				
Chronic condition	9.6%	9.0%	10.1%	0.2995
Finance				
Any financial difficulty	25.4%	25.9%	25.0%	0.5946
Low CHI coverage	15.6%	16.3%	15.0%	0.3601
Medium CHI coverage	57.4%	57.8%	57.1%	0.6850
High CHI coverage	27.0%	25.9%	27.9%	0.2305
Attitude towards risk				
Total score	76.5	81.9	72.1	0.0000
Ethical score	11.7	12.4	11.2	0.0000
Financial score	11.3	12.5	10.3	0.0000
Health/security score	13.4	14.5	12.5	0.0000
Recreational score	15.8	18.0	14.0	0.0000
Social score	24.4	24.6	24.1	0.2062
Attitude towards the future				
Self-assessed score	5.9	5.7	6.0	0.0033

^*^or vocational diploma

### Questionnaire

The questionnaire was administered by telephone and included questions all in French on (1) sociodemographic characteristics, (2) health, (3) healthcare renunciation, (4) attitude towards the future, (5) attitude towards risk, which was measured by the French version [41] of the 30-item DOSPERT scale covering five dimensions: ethical, social, health/security, financial and recreational activities [36, 37], and (6) CHI.

#### Medico-administrative data

The MGEN’s medico-administrative data were matched to the survey responses to enrich the available information on the respondents’ possible 100% coverage by the NHI for a chronic condition (the *Affection de Longue Durée* – ALD – programme) and their CHI level of coverage among the 5 possible levels.

### Variables

#### Variables to be explained

The questions on sports, tobacco and body type were formulated as follows:

- “Do you practise sports regularly (excluding journeys, do-it-yourself activities, gardening and housework)? No/Yes, less than 2 hours a week/Yes, 2 hours or more a week”;
- “Have you ever smoked? Yes, and I still smoke today/Yes, but I have stopped/No, I have never smoked”;
- “What is your height?”; “What is your weight?”.

The explained variables considered here indicated whether, at the time of the survey, the insured person (1) practised sports regularly (Y/N) regardless of the duration but excluding small daily exercises; (2) did not currently smoke (Y/N), regardless of previous smoking experience; and (3) had a normal BMI (18.5 < BMI < 25; Y/N) given his/her declared weight and height.

#### Explanatory variables

The sociodemographic variables included age, gender, marital status (couple Y/N), number of children and level of education.

The financial variables were financial difficulties Y/N and the level of CHI coverage.

A variable indicating the insured’s health status (chronic condition Y/N) was 100% coverage granted by the NHI.

Variables indicated the different scores for risk taking calculated from the answers given to the 30 items of the DOSPERT scale (i.e., the total score and five subscores corresponding to the five dimensions of the scale). The DOSPERT score was included as a covariate to proxy individual risk preferences, which were treated as stable traits directly shaping health behaviours.

A variable was also created from a visual analogue scale to measure the respondents’ attitude towards the future (from 0: *living day to day to 10: concerned about the future*) reported during the telephone interview. Time preference was included as a covariate to capture the intertemporal dimension of health behaviours, reflecting the extent to which individuals valued future health outcomes relative to immediate costs, and allowing us to disentangle its effect from that of risk attitudes.

### Statistical analysis and empirical strategy

Comparisons between subpopulations of insured persons (men and women for each health behaviour) were made using statistical tests, z tests for proportions and Student’s t tests for means.

A logistic model was estimated for each health behaviour with the previously defined explanatory variables. For each health behaviour, two models were estimated by alternatively taking the total DOSPERT score or the five subscores. More formally, the model is written as follows:

$$\:{y}_{i}^{*}={X}_{i}\beta\:+{\epsilon\:}_{i}$$

where $\:{y}_{i}^{*}$ is the unobserved latent variable for each outcome examined in the paper, $\:{X}_{i}$ the vector of values taken by the explanatory variables and $\:{\epsilon\:}_{i}$ the error term for each insured * i*. We estimated the probability to have a good health behaviour: $\:p\left({y}_{i}=1\right)=p\left({y}_{i}^{*}>0\right)=p\left({X}_{i}\beta\:>{-\epsilon\:}_{i}\right)=F\left({X}_{i}\beta\:\right),$ with * F* being the logistic cumulative distribution function.

Given the limited number of theoretically motivated specifications and the joint interpretation of coefficients within each model, we did not apply formal multiple testing corrections and instead focus on the magnitude, direction, and robustness of estimated effects.

### Heterogeneity analysis

First, each of the six baseline models (two for each health behaviour) was estimated for men and women separately. Differences in the results by gender were reported in similar studies on tobacco [33] and weight [34]. Second, for sports, tobacco and weight, the models were estimated by adopting a different definition of the explained variable. For sports, the alternative explained variable compared the respondents who participated in sports at least two hours a week with the remaining sample. Formally, $\:{y}_{i}$ was coded “1” if individual *i* performed sports at least two hours a week and “0” if individual * i* was not sporty at all or performed sports less than two hours a week. For tobacco, the explained variable was a comparison of nonsmokers who had never smoked, which excluded the 782 participants who were nonsmokers with smoking experience, and current smokers. Thus, $\:{y}_{i}$ was coded “1” if individual * i* had never smoked and “0” if individual * i* was a smoker at the time of the survey. For weight, the explained variable compared individuals who had their weight in accordance with their height (18.5 < BMI < 25) to overweight or obese individuals (BMI ≥ 25), excluding the few underweight people (BMI ≤ 18.5; *N* = 53). Here, $\:{y}_{i}$ was coded “1” if individual * i* had a normal BMI (18,5 ≤ BMI < 25) and “0” if individual * i* was either overweight (25 ≤ BMI < 30) or obese (BMI ≥ 30). Third, we estimated the models with some differences in the explanatory variables. We introduced a variable characterizing the place where the respondent lived. Two alternative definitions were tested, “size of the respondent’s municipality in inhabitants” and “category to which the respondent’s municipality belongs”, according to the French National Institute of Statistics and Economic Studies (INSEE) typology (i.e., large urban area/other urban area/other multipolar municipality/isolated municipality outside the influence of urban centres). Finally, interactions between the total DOSPERT score and age were tested.

## Results

### Prevalence of good health behaviours

**Fig. 1:**
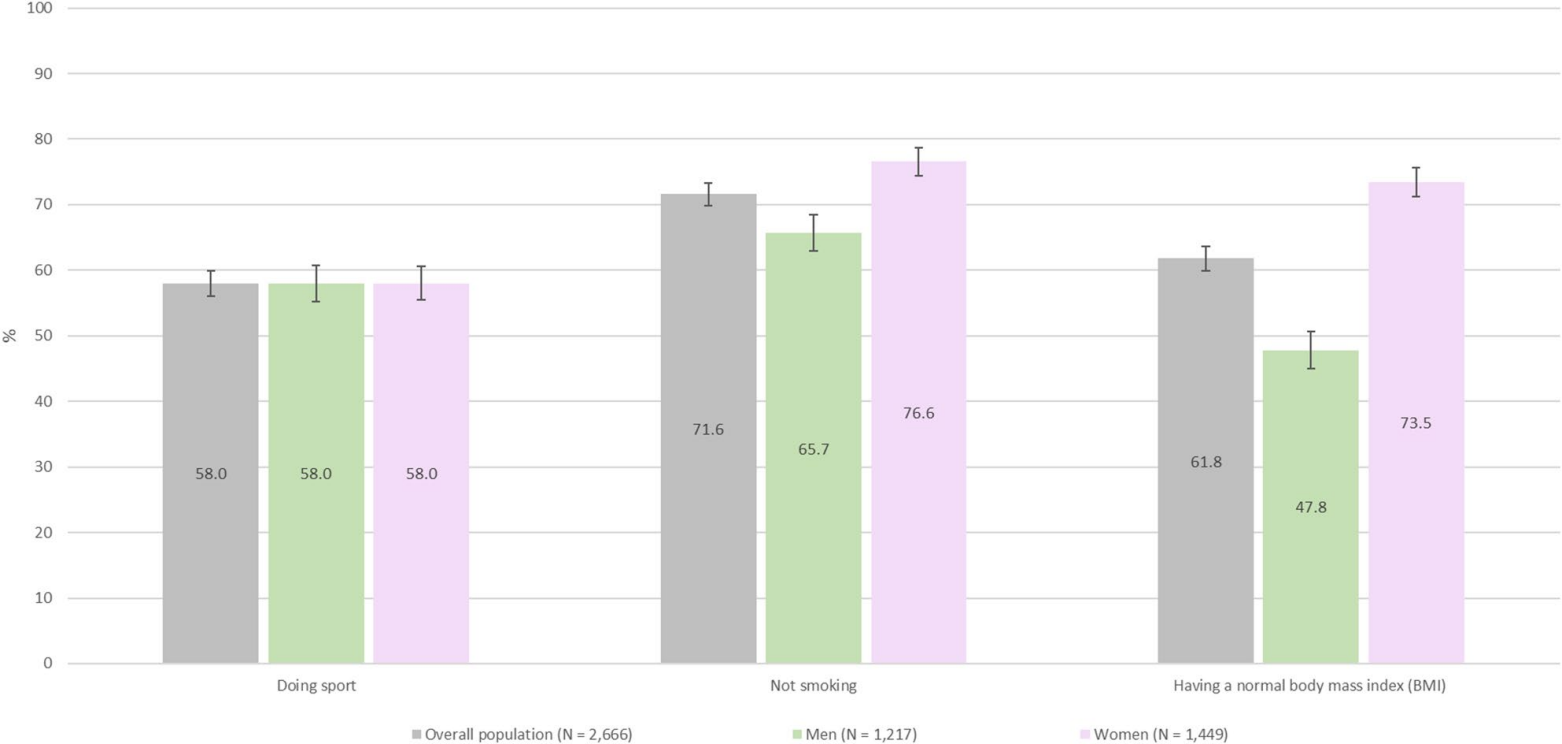
Prevalence of respondents who declared any good health behaviour For BMI, N = 2 652 (1210 men and 1442 women) as some respondents did not want to declare their weight or height

Among the 2,666 respondents, 58.0% reported participating in sports regularly, 71.6% did not smoke, and 61.8% reported having a normal BMI (Fig. 1). In addition, women were much more likely than men to have any good health behaviours except for sports, for which there was no significant difference by gender.

### Descriptive analysis

A few factors appeared to play a role in the adoption of all the health behaviours studied; however, some worked in the same direction, while others did not (Table 2). In women or individuals with a postgraduate diploma or those without financial difficulties, a greater proportion of people adopted each of the three good health behaviours, although some differences in prevalence rates were on the edge of statistical significance with a p-value just above 5%. For age and the presence of any chronic condition, statistically significant differences were found for the three health behaviours, but the signs differed. Sporty people and those with normal build were younger or had no declared chronic illnesses, whereas the opposite was true for nonsmokers.

**Table 2:** Comparison of characteristics as a percentage or average between respondents with or without any good health behaviour

	Doing sport (*N* = 2,666)			Not smoking (*N* = 2,666)			Having a normal BMI (*N* = 2,652)		
	Yes (*N* = 1,547)	No (*N* = 1,119)	*P* value	Yes (*N* = 1,910)	No (*N* = 756)	*P* value	Yes (*N* = 1,638)	No (*N* = 1,014)	*P* value
Sociodemographics									
Female	54.4%	54.3%	0.9881	58.1%	44.8%	0.0000	64.7%	37.7%	0.0000
Age	47.1	50.4	0.0000	50.5	43.5	0.0000	46.3	51.9	0.0000
Less than 36 years	27.7%	22.3%	0.0013	23.0%	31.5%	0.0000	29.5%	19.1%	0.0000
36–45 years	28.8%	26.0%	0.1157	25.3%	33.5%	0.0000	29.9%	23.9%	0.0007
46–65 years	24.8%	26.5%	0.3149	25.2%	26.4%	0.4946	23.5%	28.6%	0.0034
Over 65 years	18.7%	25.2%	0.0001	26.5%	8.6%	0.0000	17.1%	28.4%	0.0000
Living as a couple	51.1%	47.5%	0.0674	54.5%	37.4%	0.0000	48.9%	50.8%	0.3447
Number of children	1.1	1.1	0.1617	1.2	0.8	0.0000	1.1	1.2	0.0176
No university degree^*^	14.1%	24.8%	0.0000	19.4%	16.5%	0.0839	15.8%	23.1%	0.0000
Bachelor’s degree	14.0%	16.5%	0.0858	15.3%	14.4%	0.5711	14.7%	15.7%	0.4976
Undergraduate diploma	39.4%	34.6%	0.0106	35.3%	42.7%	0.0004	38.7%	35.3%	0.0787
Postgraduate diploma	32.5%	24.1%	0.0000	30.0%	26.3%	0.0590	30.8%	25.9%	0.0077
Health									
Chronic condition	8.3%	11.4%	0.0062	10.6%	7.0%	0.0043	7.5%	12.8%	0.0000
Finance									
Any financial difficulty	24.1%	27.2%	0.0736	23.1%	31.1%	0.0000	23.0%	29.3%	0.0003
Low CHI coverage	15.6%	15.5%	0.9832	14.4%	18.4%	0.0114	16.7%	13.8%	0.0441
Medium CHI coverage	57.4%	57.4%	0.9589	57.4%	57.5%	0.9400	57.5%	57.3%	0.9395
High CHI coverage	27.0%	27.1%	0.9451	28.2%	24.1%	0.0316	25.8%	28.9%	0.0825
Attitude towards risk									
Total score	81.8	69.3	0.0000	72.2	87.5	0.0000	75.9	77.6	0.1350
Ethical score	12.5	10.6	0.0000	11.0	13.5	0.0000	11.5	12.0	0.0325
Financial score	12.4	9.7	0.0000	10.4	13.5	0.0000	10.9	11.9	0.0001
Health/security score	14.1	12.4	0.0000	12.8	14.9	0.0000	13.4	13.3	0.6379
Recreational score	17.2	13.8	0.0000	14.7	18.6	0.0000	15.8	15.9	0.6003
Social score	25.5	22.8	0.0000	23.4	26.9	0.0000	24.3	24.4	0.7332
Attitude towards the future									
Self-assessed score	6.1	5.5	0.0000	5.7	6.3	0.0000	5.9	5.9	0.9224

^*^or vocational diploma

For the remaining factors, interestingly, being a couple or having more children was highly significantly associated with being a nonsmoker.

Finally, one of the most interesting findings was the contrasting results we found in relation to the DOSPERT scale for sports and tobacco, especially because they were highly significant (*p* < 0.001).

### Results of the models

Table 3 shows the results of the three logit models with the total DOSPERT score as an explanatory variable. We find that being a woman, being highly educated or having no financial difficulties may favour the adoption of good health behaviours. However, the positive marginal effect of being a woman or having no financial difficulties does not reach statistical significance for the practice of sports; in contrast, being highly educated was statistically significant for sports only.

**Table 3:** Marginal effects of factors that impact the adoption of any good health behaviour

	Doing sport (*N* = 2,666)			Not smoking (*N* = 2,666)			Having a normal BMI (*N* = 2,652)		
	Pseudo *R*² = 0.0512			Pseudo *R*² = 0.1099			Pseudo *R*² = 0.0890		
	Coef.	P value	IC 95%	Coef.	P value	IC 95%	Coef.	P value	IC 95%
Sociodemographics									
Female	0.021	0.262	-0.016 ; 0.059	0.080	0.000	0.047 ; 0.114	0.251	0.000	0.215 ; 0.288
Less than 36 years	-	-		-			-	-	
36–45 years	-0.027	0.305	-0.079 ; 0.025	-0.041	0.085	-0.088 ; 0.006	-0.040	0.097	-0.087 ; 0.007
46–65 years	-0.039	0.170	-0.096 ; 0.017	-0.013	0.617	-0.064 ; 0.038	-0.150	0.000	-0.203 ; -0.097
Over 65 years	-0.005	0.880	-0.070 ; 0.060	0.150	0.000	0.097 ; 0.203	-0.233	0.000	-0.296 ; -0.170
Living as a couple	0.010	0.583	-0.028 ; 0.049	0.128	0.000	0.093 ; 0.163	-0.041	0.028	-0.078 ; -0.004
Number of children	0.025	0.016	0.005 ; 0.045	0.015	0.120	-0.004 ; 0.034	0.010	0.299	-0.009 ; 0.029
No university degree^*^	-0.049	0.140	-0114 ; 0.016	-0.042	0.172	-0.102 ; 0.018	-0.044	0.159	-0.105 ; 0.017
Bachelor’s degree	-	-		-	-		-	-	
Undergraduate diploma	0.038	0.191	-0.019 ; 0.095	-0.010	0.692	-0.060 ; 0.040	-0.009	0.755	-0.062 ; 0.045
Postgraduate diploma	0.090	0.003	0.030 ; 0.149	0.028	0.280	-0.023 ; 0.080	0.032	0.262	-0.024 ; 0.087
Health									
Chronic condition	-0.036	0.273	-0.100 ; 0.028	0.036	0.223	-0.022 ; 0.094	-0.095	0.003	-0.158 ; -0.032
Finance									
Any financial difficulty	-0.012	0.575	-0.056 ; 0.031	-0.066	0.001	-0.105 ; -0.026	-0.046	0.033	-0.088 ; -0.004
Low CHI coverage	-0.028	0.305	-0.081 ; 0.025	0.001	0.954	-0.044 ; 0.047	0.008	0.764	-0.044 ; 0.059
Medium CHI coverage	-	-		-	-		-	-	
High CHI coverage	0.003	0.883	-0.039 ; 0.045	0.022	0.254	-0.016 ; 0.060	-0.014	0.498	-0.055 ; 0.027
Attitude towards risk									
Total score	0.003	0.000	0.003 ; 0.004	-0.002	0.000	-0.003 ; -0.002	-0.001	0.005	-0.002 ; -0.001
Attitude towards the future									
Score	0.014	0.000	0.007 ; 0.021	-0.015	0.000	-0.021 ; -0.008	-0.007	0.040	-0.014 ; -0.001

^*^or vocational diploma

Some contrasting results appear when the results are compared for the three health behaviours. The results are mixed for age and marital status for both nonsmokers and individuals with a normal BMI. Although being older or in a couple is associated with a greater likelihood of being a nonsmoker, the opposite is true for BMI, where advanced age or life as a couple decreases the chance of being of normal build. Attitude towards the future also showed contrasting results: people who were more concerned about the future had a greater likelihood of participating in sports but a lower likelihood of not smoking or having a normal BMI. Similar results were found for the attitude towards risk: individuals with a higher total DOSPERT score had a greater chance of practising sports but a lower likelihood of being either a nonsmoker or of normal build.

Interestingly, these opposite results for risk between individuals who participated in sports and those who did not smoke or who had a normal body shape were also found when the five DOSPERT subdomains were used in place of the total DOSPERT score (Table 4), although no statistical significance was reached for the risk score in ethics. A greater likelihood of practising sports was associated with a greater risk score in any of the four subdomains (i.e., ethical, financial, recreational and social) but a lower risk score in health/security. The opposite was true for either being a nonsmoker or having a normal BMI (except for the recreational score).

**Table 4:** Marginal effects of factors that impact the adoption of any good health behaviour

	Doing sport (*N* = 2,666)			Not smoking (*N* = 2,666)			Having a normal BMI (*N* = 2,652)		
	Pseudo *R*² = 0.0581			Pseudo *R*² = 0.1200			Pseudo *R*² = 0.0935		
	Coef.	P value	IC 95%	Coef.	P value	IC 95%	Coef.	P value	IC 95%
Attitude towards risk									
Ethical score	0.001	0.700	-0.004 ; 0.007	-0.003	0.264	-0.007 ; 0.002	-0.003	0.242	-0.008 ; 0.002
Financial score	0.008	0.000	0.004 ; 0.013	-0.008	0.000	-0.011 ; -0.004	-0.006	0.004	-0.010 ; -0.002
Health/security score	-0.005	0.039	-0.010 ; -0.001	0.008	0.000	0.004 ; 0.012	0.005	0.024	0.001 ; 0.010
Recreational score	0.009	0.000	0.006 ; 0.013	-0.004	0.021	-0.007 ; 0.001	0.001	0.500	-0.003 ; 0.005
Social score	0.002	0.039	0.001 ; 0.005	-0.004	0.000	-0.007 ; -0.002	-0.003	0.015	-0.005 ; -0.001

The model is adjusted on the same variables as in Table 3

### Heterogeneity analysis

#### Analysis by gender

The results of the three models stratified by gender with the total DOSPERT score are presented in Appendix 1 for men and women, respectively. Appendix 2 corresponds to the same models using the five DOSPERT subscores in place of the total DOSPERT score. Only a few changes are visible.

For the practice of sports, we find the same results as in the baseline analyses; however, the significant positive impacts of the number of children, any postgraduate diploma, and the financial or social DOSPERT scores remain for women only.

For not smoking, all the significant results identified in the baseline analysis presented in Table 3 remain for both women and men. When the DOSPERT subscores are used, some changes (compared to Table 4) appear: the significant impacts of the health/security and recreational scores persist for men only, while the significant negative effects of the financial and social scores remain for women only.

For BMI, we find the same results for men and women for age, although the marginal effect is far from significant for women between 36 and 45 years old. The significant negative effects of life as a couple, the presence of a chronic condition, the total risk score and the financial risk subscore remain for men only, whereas the negative impact of the social risk subscore persists for women only. In addition, a few effects disappear completely, such as the presence of financial difficulties, the degree of concern about the future and the health/security risk subscore, although they are close to significance for the last (*p* = 0.078 and *p* = 0.108 for women and men, respectively).

#### Alternative definitions of the variables $\:{y}_{i}$

Two models using the alternative variable $\:{y}_{i}$, presented in the methods section, are estimated for each health behaviour. These alternative variables $\:{y}_{i}$ do not change the results of the baseline multivariate analyses except a small loss of significance for some explanatory variables and a gain in significance for a few associations.

Concerning tobacco, the results are very close to significance for the number of children (the more children, the lower the likelihood of smoking). Much greater significance is observed for all age classes.

Finally, for sports, we now see a significant association between participating in sports and (1) gender, with women doing more sport than men, and (2) a young age.

#### Explanatory variables

When the place where the respondent lived was introduced into the models, no significant marginal effect was found regardless of the definition used.

#### Interaction effects

For the practice of sports, a significant interaction is found between the risk score and age when considering the overall sample, men only, or women only: as time goes by, the higher the DOSPERT score, the more likely the respondents are to be physically active.

For tobacco and BMI, there is no significant interaction effect between risk and age.

## Discussion

Our results stress the potentially important role of risk attributes in the definition of individual health behaviours and the non-monotonic contribution of those risk attributes, including when differentiated across DOSPERT scale’s domains, to a selection of health behaviours. Indeed, while individuals who do not like risk seem to have a greater likelihood of either not smoking or having a normal BMI, it appears that at the same age, risk-taking persons are more likely to be sporty. In addition, the results appear to be roughly the same between men and women when using the total DOSPERT score as an explanatory variable in the models (Appendix 1). However, gender stratification is interesting, as shown in Appendix 2, as we observe that different risk domains are at work between men and women. Finally, our results show that attitudes towards risk and the future are very different concepts that yield opposite results.

By comparing our results with the literature, we found a significant association of the same sign between attitude towards risk and tobacco or weight [32, 34]. For the latter, we found the same result for both sexes, unlike the study of Galizzi & Miraldo [34], which only found a significant association in men. In addition, we again found the well-documented protective role of a higher educational level [32] and no financial difficulties for almost all good health behaviours. Finally, it should be noted that we found a prevalence of excess weight (38%) that was slightly lower than the 47% rate recently reported for France [42].

We might wonder if a low score for risk taking could be considered a predictive factor for the adoption of good health habits. Our results show that there is a strong significant association between the respondent’s risk score and the adoption of good health habits. However, the sign of this association may vary according to the behaviour studied, suggesting that health behaviour may be determined by a general latent attitude towards risk combined with specific domain-dependent risk traits [43]. This leads individuals, whether in terms of their overall or specific level of risk aversion, to weigh up the various factors that make up their general attitude towards their health, factors which are only partially correlated with one another.

These results raise further questions. For example, first, could this information be of interest for insurers? Our study shows that, while risk attitudes provide useful information, their domain-specific and heterogeneous nature implies that they cannot, on their own, reliably predict health behaviours or guide targeted interventions. Second, while attitude towards risk is generally unobserved, are our results useful for public health stakeholders? The individual attitude towards risk considered in this article may affect behaviours that are linked to the development of diseases, the importance of which has been amply demonstrated in the literature, both in terms of the population concerned and the health costs involved [44]. On the one hand, our results are useful for awareness campaigns because they show that it is necessary to revise a discourse that is generally the same for everyone and for all health behaviours. On the other hand, physicians should also adapt their words to their patients’ attitude towards risk.

The strengths of this article are that it relies on an original phone survey that (1) documented a quite comprehensive set of individual characteristics, which limits the magnitude of unobserved heterogeneity as usual in this kind of framework, and (2) used a questionnaire, including a validated 30-item instrument for risk-taking measurement, which enabled the study of domain subscores.

Some limitations, however, exist. First, because of the cross-sectional nature of the dataset, we are highlighting associations, not causal relationships. For example, people who play sport may score higher on the DOSPERT scale because sport has trained them to take risks, not because risk-takers are drawn to sport. In this regard, distinguishing between the different domains of the DOSPERT score makes it possible to disentangle the potentially contrasting manifestations of attitude towards risk. Second, the DOSPERT score may be sensitive to the individual context that we do not know otherwise. Other instruments could have been used to measure attitude towards risk, such as lotteries. However, lotteries are difficult to use in population-based studies, which is why they are often used in specific and/or insider populations (e.g., students). Moreover, one study showed that lotteries are unable to predict risky behaviours in the domain of health [45]. Third, we should have asked a question to characterize the sport practised, as there may be differences between, caricaturally, the aquagym and the ultratrail. Fourth, if we had a larger sample size, it would have been interesting to study people with a “healthy” profile that combined at least two good health behaviours, such as not smoking and having a normal BMI, in order to reveal any potential spillover effects from one heath habit to another one. Fifth, although our questionnaire included a large amount of information, we cannot exclude the existence of unobserved heterogeneity, particularly in the case of BMI, as weight problems are multifactorial. Sixth, the respondents were selected at random from CHI subscribers. Therefore, we studied a distribution conditional on having subscribed to a CHI contract, which might correspond to a specific part of the French population with regard to attitude towards risk. However, CHI subscribers represent almost the entire French population (95%) [40]. Among them, people who are risk-averse take out insurance to limit the financial consequences of adverse events and adopt preventive measures to reduce the likelihood of such events [46–49]. But, it remains yet unclear whether risk avoiders or risk takers are overrepresented in this sample. Seventh, we cannot rule out the usual caveat regarding reported data on height and weight. Indeed, a large body of evidence, including recent systematic reviews [50], shows that self-reported anthropometric measures are affected by systematic biases – height being generally overestimated and weight underestimated – resulting in a downward bias in BMI and misclassification of overweight and obesity status. Finally, the MGEN is known for having covered historically civil servants, mainly teachers, and their relatives. This population may appear to be more sensitive to health issues. Yet, the CHI contract under study is open to all, which tends to limit this bias.

## Conclusion

Risk aversion has an inherently ambiguous relationship with health behaviours: individuals allocate risk-reducing effort across domains subject to preferences, constraints, and adjustment costs, so that more demanding changes – such as engaging in regular physical activity – are less readily adopted than lower-effort behaviours – such as abstaining from smoking when one has never smoked – leading to uneven and sometimes compensatory patterns. This ambiguity is further highlighted by the multidimensional nature of risk attitudes captured by the DOSPERT scale, where health-related risk aversion may exert a different influence than social or ethical risk aversion, for instance. Consistent with this perspective, our empirical results point to heterogeneous and domain-specific associations between risk attitudes and health behaviours rather than a uniform relationship. These findings suggest that policies or interventions based solely on aggregate measures of risk aversion may have limited effectiveness, and that a more nuanced, behaviour-specific approach is required to better account for the complexity of individual decision-making.

## Supplementary Information

Supplementary Material 1.

## Data Availability

The French data protection authority (CNIL) demands strict compliance with data protection and confidentiality requirements.
