# Supplementary material for: What does attitude towards risk tell us about the adoption of good health behaviours? Results from a telephone survey
Source: BMC Public Health. 2026 Jun 11;26:2381. doi: 10.1186/s12889-026-27960-7 (PMC13483120; doi:10.1186/s12889-026-27960-7)
Supplement: Supplementary file 1 [file 12889_2026_27960_MOESM1_ESM.docx]

Appendix 1: Marginal effects of factors that impact the adoption of any good health behaviour by gender

|  | Doing sport | | | | | | |
| --- | --- | --- | --- | --- | --- | --- | --- |
|  | Women  (N =1,449) | | |  | Men  (N = 1,217) | | |
|  | Pseudo R² = 0.0503 | | |  | Pseudo R² = 0.0736 | | |
|  | Coef. | P value | IC 95% |  | Coef. | P value | IC 95% |
| Sociodemographics |  |  |  |  |  |  |  |
| Less than 36 years | - | - |  |  | - | - |  |
| 36-45 years | -0.037 | 0.300 | -0.107 ; 0.033 |  | -0.011 | 0.769 | -0.088 ; 0.065 |
| 46-65 years | -0.065 | 0.099 | -0.142 ; 0.012 |  | -0.026 | 0.538 | -0.109 ; 0.057 |
| Over 65 years | 0.005 | 0.921 | -0.084 ; 0.093 |  | 0.001 | 0.983 | -0.095 ; 0.097 |
| Living as a couple | 0.023 | 0.393 | -0.030 ; 0.076 |  | 0.010 | 0.732 | 0.046 ; 0.066 |
| Number of children | 0.043 | 0.002 | 0.015 ; 0.071 |  | 0.0003 | 0.986 | -0.029 ; 0.030 |
| No university degree* | -0.122 | 0.010 | -0.216 ; -0.029 |  | 0.012 | 0.791 | -0.078 ; 0.102 |
| Bachelor’s degree | - | - |  |  | - | - |  |
| Undergraduate diploma | -0.003 | 0.941 | -0.081 ; 0.075 |  | 0.088 | 0.038 | 0.005 ; 0.172 |
| Postgraduate diploma | 0.119 | 0.004 | 0.037 ; 0.200 |  | 0.049 | 0.265 | -0.037 ; 0.134 |
| Health |  |  |  |  |  |  |  |
| Chronic condition | -0.011 | 0.794 | -0.095 ; 0.073 |  | -0.055 | 0.274 | -0.155 ; 0.044 |
| Finance |  |  |  |  |  |  |  |
| Any financial difficulty | 0.056 | 0.059 | -0.002 ; 0.115 |  | -0.093 | 0.004 | -0.156 ; -0.029 |
| Low CHI coverage | -0.026 | 0.481 | -0.100 ; 0.047 |  | -0.022 | 0.578 | -0.098 ; 0.055 |
| Medium CHI coverage | - | - |  |  | - | - |  |
| High CHI coverage | 0.001 | 0.975 | -0.056 ; 0.058 |  | -0.004 | 0.905 | -0.066 ; 0.059 |
| Attitude towards risk |  |  |  |  |  |  |  |
| Total score | 0.003 | 0.000 | 0.002 ; 0.004 |  | 0.004 | 0.000 | 0.003 ; 0.005 |
| Attitude towards the future |  |  |  |  |  |  |  |
| Score | 0.012 | 0.013 | 0.003 ; 0.022 |  | 0.015 | 0.004 | 0.005 ; 0.025 |

*or vocational diploma

|  | Not smoking | | | | | | |
| --- | --- | --- | --- | --- | --- | --- | --- |
|  | Women  (N = 1,449) | | |  | Men  (N = 1,217) | | |
|  | Pseudo R² = 0.1404 | | |  | Pseudo R² = 0.0944 | | |
|  | Coef. | P value | IC 95% |  | Coef. | P value | IC 95% |
| Sociodemographics |  |  |  |  |  |  |  |
| Less than 36 years | - | - |  |  | - | - |  |
| 36-45 years | -0.018 | 0.547 | -0.078 ; 0.041 |  | -0.066 | 0.079 | -0.139 ; 0.008 |
| 46-65 years | -0.007 | 0.838 | -0.073 ; 0.059 |  | -0.001 | 0.973 | -0.082 ; 0.079 |
| Over 65 years | 0.128 | 0.000 | 0.061 ; 0.196 |  | 0.183 | 0.000 | 0.097 ; 0.269 |
| Living as a couple | 0.158 | 0.000 | 0.113 ; 0.202 |  | 0.091 | 0.001 | 0.037 ; 0.146 |
| Number of children | 0.0003 | 0.979 | -0.024 ; 0.024 |  | 0.036 | 0.020 | 0.006 ; 0.066 |
| No university degree* | -0.160 | 0.000 | -0.239 ; -0.073 |  | 0.063 | 0.167 | -0.026 ; 0.151 |
| Bachelor’s degree | - | - |  |  | - | - |  |
| Undergraduate diploma | -0.021 | 0.515 | -0.083 ; 0.041 |  | -0.0006 | 0.988 | -0.081 ; 0.079 |
| Postgraduate diploma | 0.025 | 0.450 | -0.040 ; 0.090 |  | 0.022 | 0.596 | 0.060 ; 0.104 |
| Health |  |  |  |  |  |  |  |
| Chronic condition | -0.010 | 0.772 | -0.080 ; 0.059 |  | 0.121 | 0.014 | 0.025 ; 0.218 |
| Finance |  |  |  |  |  |  |  |
| Any financial difficulty | -0.047 | 0.058 | -0.096 ; 0.002 |  | -0.078 | 0.015 | -0.140 ; -0.015 |
| Low CHI coverage | 0.012 | 0.693 | -0.046 ; 0.069 |  | -0.008 | 0.826 | -0.078 ; 0.062 |
| Medium CHI coverage | - | - |  |  | - | - |  |
| High CHI coverage | 0.026 | 0.271 | -0.021 ; 0.073 |  | 0.005 | 0.884 | -0.056 ; 0.065 |
| Attitude towards risk |  |  |  |  |  |  |  |
| Total score | -0.004 | 0.000 | -0.004 ; -0.003 |  | -0.001 | 0.037 | -0.002 ; -0.001 |
| Attitude towards the future |  |  |  |  |  |  |  |
| Score | -0.013 | 0.002 | -0.021 ; 0.005 |  | -0.017 | 0.002 | -0.027 ; -0.006 |

*or vocational diploma

|  | Having a normal BMI | | | | | | |
| --- | --- | --- | --- | --- | --- | --- | --- |
|  | Women  (N = 1,442) | | |  | Men  (N = 1,210) | | |
|  | Pseudo R² = 0.0431 | | |  | Pseudo R²= 0.0509 | | |
|  | Coef. | P value | IC 95% |  | Coef. | P value | IC 95% |
| Sociodemographics |  |  |  |  |  |  |  |
| Less than 36 years | - | - |  |  | - | - |  |
| 36-45 years | -0.003 | 0.925 | -0.061 ; 0.055 |  | -0.073 | 0.066 | -0.150 ; 0.005 |
| 46-65 years | -0.123 | 0.000 | -0.191 ; -0.055 |  | -0.178 | 0.000 | -0.263 ; -0.094 |
| Over 65 years | -0.191 | 0.000 | -0.276 ; -0.107 |  | -0.261 | 0.000 | -0.357 ; -0.164 |
| Living as a couple | 0.015 | 0.539 | -0.033 ; 0.062 |  | -0.110 | 0.000 | -0.167 ; -0.052 |
| Number of children | 0.013 | 0.292 | -0.011 ; 0.038 |  | 0.011 | 0.499 | -0.020 ; 0.041 |
| No university degree* | -0.055 | 0.205 | -0.140 ; 0.030 |  | -0.014 | 0.764 | -0.106 ; 0.078 |
| Bachelor’s degree | - | - |  |  | - | - |  |
| Undergraduate diploma | 0.030 | 0.405 | -0.040 ; 0.100 |  | -0.039 | 0.373 | -0.124 ; 0.047 |
| Postgraduate diploma | 0.053 | 0.162 | -0.021 ; 0.127 |  | 0.039 | 0.383 | -0.048 ; 0.126 |
| Health |  |  |  |  |  |  |  |
| Chronic condition | -0.030 | 0.430 | -0.104 ; 0.044 |  | -0.182 | 0.000 | -0.280 ; -0.084 |
| Finance |  |  |  |  |  |  |  |
| Any financial difficulty | -0.042 | 0.133 | -0.096 ; 0.013 |  | -0.048 | 0.153 | -0.113 ; 0.018 |
| Low CHI coverage | 0.026 | 0.457 | -0.046 ; 0.057 |  | -0.011 | 0.772 | -0.089 ; 0.066 |
| Medium CHI coverage | - | - |  |  | - | - |  |
| High CHI coverage | 0.006 | 0.831 | -0.046 ; 0.057 |  | -0.045 | 0.177 | -0.109 ; 0.020 |
| Attitude towards risk |  |  |  |  |  |  |  |
| Total score | -0.0001 | 0.840 | -0.001 ; 0.001 |  | -0.002 | 0.001 | -0.002 ; -0.001 |
| Attitude towards the future |  |  |  |  |  |  |  |
| Score | -0.006 | 0.193 | -0.014 ; 0.003 |  | -0.007 | 0.192 | -0.018 ; 0.004 |

*or vocational diploma

Appendix 2: Marginal effects of factors impacting the adoption of any good health behaviour by gender

|  | Doing sport | | | | | | |
| --- | --- | --- | --- | --- | --- | --- | --- |
|  | Women  (N = 1,449) | | |  | Men  (N = 1,217) | | |
|  | Pseudo R² = 0.0580 | | |  | Pseudo R² = 0.0881 | | |
|  | Coef. | P value | IC 95% |  | Coef. | P value | IC 95% |
| Attitude towards risk |  |  |  |  |  |  |  |
| Ethical score | -0.006 | 0.150 | -0.013 ; 0.002 |  | 0.008 | 0.043 | 0.001 ; 0.016 |
| Financial score | 0.013 | 0.000 | 0.007 ; 0.020 |  | 0.004 | 0.189 | -0.002 ; 0.010 |
| Health/security score | -0.003 | 0.346 | -0.100 ; 0.003 |  | -0.006 | 0.110 | -0.012 ; 0.001 |
| Recreational score | 0.006 | 0.030 | 0.001 ; 0.012 |  | 0.013 | 0.000 | 0.008 ; 0.018 |
| Social score | 0.005 | 0.001 | 0.002 ; 0.008 |  | -0.002 | 0.374 | -0.005 ; 0.002 |

The model is adjusted on the same variables as in Table 3.

|  | Not smoking | | | | | | |
| --- | --- | --- | --- | --- | --- | --- | --- |
|  | Women  (N = 1,449) | | |  | Men  (N = 1,217) | | |
|  | Pseudo R² = 0.1563 | | |  | Pseudo R² = 0.1109 | | |
|  | Coef. | P value | IC 95% |  | Coef. | P value | IC 95% |
| Attitude towards risk |  |  |  |  |  |  |  |
| Ethical score | 0.001 | 0.646 | -0.005 ; 0.007 |  | -0.005 | 0.197 | -0.011 ; 0.002 |
| Financial score | -0.014 | 0.000 | -0.019 ; -0.009 |  | -0.001 | 0.668 | -0.006 ; 0.004 |
| Health/security score | 0.001 | 0.656 | -0.004 ; 0.006 |  | 0.014 | 0.000 | 0.007 ; 0.020 |
| Recreational score | 0.001 | 0.798 | -0.004 ; 0.005 |  | -0.010 | 0.000 | -0.015 ; -0.005 |
| Social score | -0.007 | 0.000 | -0.009 ; -0.004 |  | -0.001 | 0.780 | -0.004 ; 0.003 |

The model is adjusted on the same variables as in Table 3.

|  | Having a normal BMI | | | | | | |
| --- | --- | --- | --- | --- | --- | --- | --- |
|  | Women  (N = 1,442) | | |  | Men  (N = 1,210) | | |
|  | Pseudo R² = 0.0487 | | |  | Pseudo R² = 0.0600 | | |
|  | Coef. | P value | IC 95% |  | Coef. | P value | IC 95% |
| Attitude towards risk |  |  |  |  |  |  |  |
| Ethical score | -0.003 | 0.378 | -0.010 ; 0.004 |  | -0.007 | 0.076 | -0.015 ; 0.001 |
| Financial score | -0.001 | 0.675 | -0.007 ; 0.005 |  | -0.008 | 0.006 | -0.014 ; -0.002 |
| Health/security score | 0.006 | 0.078 | -0.001 ; 0.012 |  | 0.006 | 0.108 | -0.001 ; 0.013 |
| Recreational score | 0.003 | 0.233 | -0.002 ; 0.008 |  | 0.001 | 0.831 | -0.005 ; 0.006 |
| Social score | -0.003 | 0.011 | -0.006 ; -0.001 |  | -0.002 | 0.312 | -0.006 ; 0.002 |

The model is adjusted on the same variables as in Table 3.
